# In Vitro Effects of Lipopolysaccharide on Rabbit Sperm: Toll-like Receptor 4 Expression, Motility, and Oxidative Status

**DOI:** 10.3390/antiox14040431

**Published:** 2025-04-02

**Authors:** Alda Quattrone, Nour Elhouda Fehri, Stella Agradi, Laura Menchetti, Olimpia Barbato, Marta Castrica, Majlind Sulçe, Cesare Castellini, Gerald Muça, Simona Mattioli, Daniele Vigo, Giovanni Migni, Lorenzo Nompleggio, Rafik Belabbas, Fabio Gualazzi, Giovanni Ricci, Rezart Postoli, Francesca Di Federico, Elena Moretti, Pellumb Zalla, Giulia Collodel, Gabriele Brecchia, Giulio Curone

**Affiliations:** 1 Department of Veterinary Medicine and Animal Sciences, University of Milan, Via dell’Università 6, 26900 Lodi, Italy; alda.quattrone@unimi.it (A.Q.); nour.fehri@unimi.it (N.E.F.); daniele.vigo@unimi.it (D.V.); gabriele.brecchia@unimi.it (G.B.); giulio.curone@unimi.it (G.C.); 2 Department of Veterinary Sciences, University of Torino, Largo Paolo Braccini 2, 10095 Grugliasco, Italy; 3 School of Biosciences and Veterinary Medicine, University of Camerino, Via Circonvallazione 93/95, 62024 Matelica, Italy; fabio.gualazzi@studenti.unicam.it; 4 Department of Veterinary Medicine, University of Perugia, Via San Costanzo 4, 06126 Perugia, Italy; olimpia.barbato@unipg.it (O.B.); giovanni.ricci@unipg.it (G.R.); 5 Department of Comparative Biomedicine and Food Science, University of Padova, Viale dell’Università 16, 35020 Legnaro, Italy; marta.castrica@unipd.it; 6 Faculty of Veterinary Medicine, Agricultural University of Tirana, Kodër Kamëz, 1029 Tirana, Albania; msulce@ubt.edu.al (M.S.); gmuca@ubt.edu.al (G.M.); rezart.postoli@ubt.edu.al (R.P.); pellumb.zalla@ubt.edu.al (P.Z.); 7 Department of Agricultural, Food and Environmental Science, University of Perugia, Borgo XX Giugno 74, 06124 Perugia, Italy; cesare.castellini@unipg.it (C.C.); simona.mattioli@unipg.it (S.M.); giovanni.migni@unipg.it (G.M.); lorenzo.nompleggio@studenti.unipg.it (L.N.); francesca.difederico@unipg.it (F.D.F.); 8 Laboratory of Research “Health and Animal Productions”, Higher National Veterinary School, Road Issad 26 Abes, Oued Smar, Algiers 16200, Algeria; r.belabbas@ensv.dz; 9 Department of Molecular and Developmental Medicine, University of Siena, 53100 Siena, Italy; elena.moretti@unisi.it (E.M.); giulia.collodel@unisi.it (G.C.)

**Keywords:** rabbit buck, sperm motility, in vitro, CASA system, lipopolysaccharide, toll-like receptor, TLR4, oxidative stress, malondialdehyde, male infertility

## Abstract

Lipopolysaccharide (LPS)-induced inflammation impairs sperm function; however, its impact on ejaculated rabbit sperm remains unexplored. This dose-response study aims to determine the LPS concentration that negatively affects sperm motility in vitro, while also providing the first identification of TLR4 localization on rabbit spermatozoa. Additionally, it evaluates malondialdehyde (MDA) levels in seminal plasma as an indicator of oxidative stress. Sperm motility was analyzed using computer-assisted sperm analysis (CASA) after incubation with increasing LPS concentrations (0, 50, 100, 200, 400, 600, and 800 µg/mL) at multiple time points (0, 1, 2, and 4 h). LPS doses ≥ 400 µg/mL significantly reduced progressive and non-progressive motility, as well as curvilinear velocity (all *p* < 0.001), while increasing the proportion of static spermatozoa (*p* < 0.05). Receiver operating characteristic (ROC) analysis identified 300 µg/mL as the threshold dose for motility decline. Immunofluorescence revealed TLR4 localization in the midpiece of sperm tails, with weak labeling in control samples and a marked increase after 4 h of incubation with 400 μg/mL LPS. MDA levels were assessed using the thiobarbituric acid reactive substances (TBARS) assay with a colorimetric kit, showing no significant effect of LPS treatment. No correlation was found between MDA and other semen parameters. ccThese findings identify TLR4 on rabbit sperm for the first time and establish a threshold LPS dose for future in vitro studies.

## 1. Introduction

In both humans and animals, one of the leading causes of male infertility is the inflammation of the genital tract [1], which is often due to infections caused by pathogenic microorganisms [2]. In livestock animals, including rabbits, clinical and subclinical infections of the genital tract not only pose a significant welfare concern but also lead to substantial economic losses [3]. These losses arise from reduced productivity, increased culling rates, and expenses for medications and veterinary services [4,5]. Genital infections in rabbits, as well as in humans and other animal species, are commonly associated with Gram-negative bacteria such as *Escherichia coli*, *Pasteurella multocida*, *Pseudomonas* spp., and *Salmonella* spp. [6,7]. These pathogens can compromise the reproductive function by triggering inflammatory responses and causing damage to sperm quality [8]. Indeed, both acute and chronic inflammatory conditions negatively impact testicular spermatogenesis and steroidogenesis, leading to reduced sperm motility, viability [9,10], and overall male reproductive function [11].

Endotoxins such as lipopolysaccharide (LPS), a crucial component of the outer membrane of Gram-negative bacteria, are widely used to simulate both in vivo and in vitro inflammation in several organs and cell cultures [12,13] and, therefore, could be used to study the mechanisms by which infections and/or inflammation impair the reproductive system [6]. In vivo studies have demonstrated that LPS can negatively affect sperm maturation and quality both directly by binding to cells of the reproductive tract and indirectly by stimulating immune cells [14]. LPS exerts its effects in vivo by inducing an inflammatory response through the interaction with Toll-like receptor 4 (TLR4), a pattern recognition receptor expressed in cells of the immune system and the reproductive tract [6]. TLR4 plays a central role in the innate immune system by recognizing pathogen-associated molecular patterns (PAMPs), such as LPS, and initiating signaling pathways that lead to the production of pro-inflammatory cytokines and chemokines [15]. In the genital tract of the male rabbit, TLR4 is predominantly expressed in the testis, epididymis, and seminal vesicles [16]. The binding of LPS to TLR4 stimulates the release of pro-inflammatory cytokines, which subsequently promote the production of oxidative mediators such as reactive oxygen species (ROS) and nitric oxide (NO) [17]. These oxidative mediators are believed to contribute to structural, metabolic, and functional impairments in Sertoli and germ cells, decreased testosterone levels, and alterations in semen quality and traits [17].

While the mechanisms of LPS-induced damage in vivo have been well-documented in rabbits, its in vitro effects, particularly the underlying mechanisms, along with the presence and localization of TLR4 on rabbit spermatozoa, remain largely unexplored. In vitro studies offer valuable insights into the mechanisms occurring in vivo, with the advantage of avoiding the induction of an inflammatory response in live animals. Furthermore, in vitro experiments using LPS on animal sperm enable direct observation of its effects on sperm function under controlled conditions, minimizing the variability associated with in vivo systems [18]. However, no established protocol exists for determining the appropriate LPS dose in rabbits, where no relevant literature is available. Rabbits are an ideal animal model for investigating LPS effects on the reproductive system due to the ease and non-invasive nature of semen collection [3,19]. In this species, repeated semen collection using an artificial vagina without sacrificing the animal provides significant ethical and practical advantages over other models, such as mice and rats, where such methods are not feasible [3].

In vitro studies on humans and various animal species have demonstrated that LPS affects sperm function in a dose- and time-dependent manner, with significant impacts on motility and viability, through various mechanisms of action [18,19,20]. The detrimental effects of LPS in vitro on sperm are primarily attributed to the excessive production of ROS. This LPS-induced oxidative stress can lead to lipid peroxidation of sperm membranes [10,21], disrupting their structural integrity and impairing motility [10]. A key indicator of lipid peroxidation is malondialdehyde (MDA), which is commonly measured using the thiobarbituric acid reactive substances (TBARS) assay. Elevated levels of MDA have been associated with decreased sperm motility and increased morphological abnormalities, indicating oxidative damage to sperm cells [22].

This study investigates the impact of lipopolysaccharide (LPS) on rabbit sperm motility in a dose- and time-dependent manner, identifying a threshold concentration at which motility significantly declines. We hypothesize that these effects are mediated through TLR4 receptor activation and oxidative stress pathways. Specifically, this study aims to: (i) determine the LPS concentration that impairs sperm motility in vitro; (ii) identify TLR4 on rabbit spermatozoa for the first time; and (iii) evaluate MDA levels in seminal plasma as an indicator of oxidative stress.

These findings will inform future research on LPS action, potential mitigation strategies and the link between bacterial infections and male infertility with implications for both rabbits and humans.

## 2. Materials and Methods

### 2.1. Animals and Experimental Design

The animal trial was conducted at the experimental farm of the Department of Agricultural, Food and Environmental Science of the University of Perugia (Italy). The Italian Ministry and the Ethical Committee of the University of Perugia (Authorization n° 325/2021-PR) approved the experimental protocol. This study was carried out in accordance with the EU Directive 2010/63 for the protection of animals used for scientific purposes.

Six New Zealand White rabbit bucks of the same age (8 months) and weight (approximately 4 kg) were housed in individual cages under controlled environmental conditions for temperature (18–21 °C) and relative humidity (60%), with a photoperiod of 16 h of light and 8 h of darkness. The rabbits were fed ad libitum with a standard diet [23], and fresh water was always available. All rabbit bucks were trained to use the artificial vagina, and their clinical status, health, and welfare were closely monitored.

**Figure 1:**
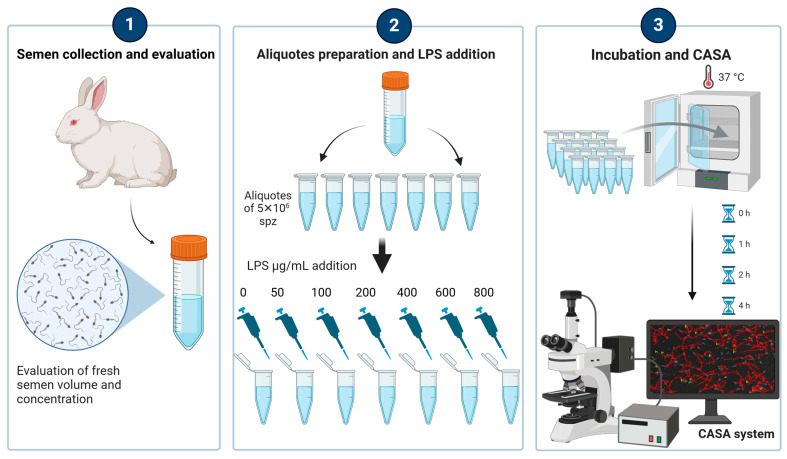
Experimental design: Semen was collected using an artificial vagina from six rabbit bucks and immediately evaluated for volume and concentration. In the laboratory, the semen sample from each rabbit was divided into seven aliquots, each containing 5 × 10^6^ spermatozoa. Each aliquot was then treated with escalating doses of lipopolysaccharide (LPS) at concentrations of 0, 50, 100, 200, 400, 600, and 800 µg/mL. To ensure accuracy and reproducibility, three replicates were performed for each aliquot, resulting in a total of 504 assessments. Sperm motility was assessed using a computer-assisted sperm analysis (CASA) system at the following time points: immediately after LPS inoculation (TpostLPS), and after 1 h (T1h), 2 h (T2h), and 4 h (T4h) of incubation. All treatments and evaluations were performed under consistent environmental and laboratory conditions to ensure the reliability and reproducibility of the results.

To determine the effective dose of LPS for the induction of measurable changes in sperm motility in vitro, we used LPS from *Escherichia coli* serotype O127:B8 (Sigma–Aldrich, Steffeld, Germany), which has been reported to induce inflammation in vivo in both male and female rabbits [6]. The semen sample obtained from each rabbit was divided into seven aliquots, which were respectively added with escalating doses of LPS (0, 50, 100, 200, 400, 600, and 800 µg/mL). For each aliquot (*n* = 42), three replicates were performed (*n* = 126), and sperm qualitative parameters were monitored immediately after inoculation with LPS (TpostLPS) and after 1 (T1h), 2 (T2h), and 4 (T4h) h of incubation (Figure 1). In total, 504 assessments were carried out. By using samples without LPS (i.e., 0 µg/mL) as a control and increasing LPS concentrations, while evaluating sperm response over time, we aimed to establish a dose-dependent threshold at which significant changes in sperm motility occurred.

### 2.2. Semen Collection and Analysis

Semen was collected from each rabbit buck using a teaser doe and an artificial vagina preheated to 38 °C with water. The semen samples were then macroscopically evaluated, and only those displaying a homogenous white opalescent color were included in the experiment, while those containing urine, cell debris, or lipid droplets were excluded from further analysis [24]. Pasteur pipettes were used to remove gel plugs if present. After collection, semen samples were immediately transported to the laboratory within 15 min, maintained at a temperature of around 27 °C. Once in the laboratory, semen volume (mL) and sperm concentration (10^6^ spermatozoa/mL) were measured according to the guidelines of the International Rabbit Reproduction Group [24] (Boiti et al., 2005). A Thoma-Zeiss cell counting chamber and a light microscope (Olympus CH-2, Olympus Corporation, Tokyo, Japan) set at 400× objective magnification were used for measuring sperm concentration with a direct cell count method, following the equation (Equation (1)):

Spermatozoa Concentration (10^6^/mL) = (N_spz_/32) × 100 (1)

To generate multiple replicates, each raw semen sample was divided into aliquots containing 5 × 10^6^ spermatozoa. These aliquots were then diluted (1:5) with modified Tyrode’s Albumin Lactate Pyruvate (TALP) medium. TALP medium (Sigma Chemical Co. Milan, Italy) was composed of NaCl (5.69 g/L), KCl (0.23 g/L), CaCl_2_·2H_2_O (0.29 g/L), MgCl_2_·6H_2_O (0.08 g/L), Na_2_HPO_4_ (0.04 g/L), NaHCO_3_ (2.09 g/L), Na pyruvate (0.02 g/L), lactic acid (0.37%, *v*/*v*), HEPES (2.38 g/L), gentamicine (50 mg/L), and BSA (0.30%). Its osmolarity and pH values were 296 mOsm/kg and 7.4, respectively.

After dilution, the aliquots of each rabbit were incubated at 37 °C in a 5% of CO_2_ atmosphere with the different doses (i.e., 0/control, 50, 100, 200, 400, 600, and 800 μg/mL) of lipopolysaccharide (LPS) derived from *Escherichia coli* (mother solution 1 mg/mL of LPS O127:B8, Sigma–Aldrich, Steffeld, Germany).

The kinematic parameters were analyzed at each time point (i.e., TpostLPS, T1h, T2h, and T4h) with a computer-assisted sperm analysis (CASA) system (ISAS^®^ model ISASv1 Proiser RD, S.L, Valencia, Spain) equipped with an HS640C video camera. The setup parameters were the same as those defined in previous experiments [25]. A 10 μL drop from each aliquot of semen was placed on a Makler chamber that was prewarmed at 37 °C. The microscope’s heated stage was consistently maintained at 37 °C throughout the analysis to ensure stable and optimal conditions. For each sample, at least six microscopic fields were analyzed for a minimum of 300 sperm tracks. All semen samples were recorded at 100 Hz frames for 1 s; thus, 12–200 successive images were recorded. The kinematic parameters recorded were the following: % of static sperms, progressive motility (%), non-progressive motility (%), curvilinear velocity (VCL, μm/s), straight line velocity (VS.L, μm/s), average path velocity (VAP, μm/s), linearity (LIN, %), straightness (STR, %), amplitude of lateral head displacement (ALH, μm), and beat-cross frequency (BCF, Hz) [26]. Among these parameters, static sperm, progressive motility, non-progressive motility, curvilinear velocity, linearity, ALH, and BCF are detailed in Table 1, while VS.L, VAP, and STR are provided in Table S1.

### 2.3. Measurement of Lipid Peroxidation: Malondialdehyde (MDA) Assay

From the original aliquots of semen samples treated with escalating doses of LPS, 92 were randomly selected to assess MDA levels. The MDA/TBARS in semen samples were evaluated using a multispecies colorimetric assay kit (Zx-44116-96, ZellBio Gmbh, Berlin, Germany). Malondialdehyde (MDA) concentration was measured by the determination of the optical density at 535 nm using a Spectrophotometer Tecan Infinit Pro 200 (Tecan Trading AG, Männedorf, Switzerland). The analytical sensitivity was 0.36 micromoles (μM) and the intra-assay and inter-assay coefficients were 3.5% and 4.5%, respectively.

### 2.4. Expression of Toll-like Receptor-4 in Rabbit Sperm with Immunofluorescence Staining

Fresh semen samples from all rabbits were used to evaluate TLR4 expression. Immunofluorescence was performed on each sample to examine TLR4 localization in rabbit spermatozoa. This analysis was conducted both in the absence of LPS (control) and after a 4-h incubation with 400 μg/mL LPS, the dose that significantly reduced sperm motility. Rabbit-smeared spermatozoa were treated with PBS containing serum albumin (BSA) 1% and normal goat serum (NGS) 5% for 20 min at room temperature. Then, the slides were incubated overnight at 4 °C in a humid chamber with the primary antibodies: CD284 (TLR4) Monoclonal Antibody (HTA125) eBioscence, (Invitrogen, Thermo Fisher Scientific, Carlsbad, CA, USA) diluted 1:200. The reaction was revealed by goat anti-mouse antibody conjugated to Alexa Fluor 488 (Invitrogen, Thermo Fisher Scientific, Carlsbad, CA, USA), diluted at 1:500; the incubation was performed at room temperature for 1 h. In control, the primary antibody was omitted. Nuclei were stained with 4,6-diamidino-2-phenylindole (DAPI) solution (Vysis, Downers Grove, IL, USA). Slides were analyzed with a Leica DMI 6000 Fluorescence Microscope (Leica Microsystems GmbH, Wetzlar, Germany), and the images were acquired by Leica AF6500 Integrated System for Imaging and Analysis (Leica Microsystems, Germany). At least 200 spermatozoa were scored for each sperm sample.

### 2.5. Statistical Analysis

Diagnostic graphs were used to check assumptions and identify outliers. Logarithmic transformation was used for static spermatozoa, while quadratic transformation was used for non-progressive motility and ALH. The raw data are reported in the tables as means and standard errors (SEs). An outlier was eliminated among the TBARS values (>15.00 μM). Qualitative data were then analyzed by Linear Mixed Models (LMMs), where replicate (3 for each sample), LPS Dose (7 levels, 0–800 μg/mL), and Time (TpostLPS, T1h, T2h, and T4h) were included as the within-subject effect with a compound symmetry structure and with the rabbit as a random factor. The LMMs evaluated the main effects of dose, time, and interaction. The Sidak correction was used for multiple comparisons. Then, for each parameter, the optimal LPS dose to induce spermatic damage was determined. First, the marginal means of the control group (i.e., LPS Dose = 0 μg/mL) during the observation period were calculated and were used to define the acceptability limit for each parameter according to Equation (2) [27]:

(2) Marginal mAcceptability limit=eans−Standard deviation

Second, receiver operating characteristic (ROC) analyses were performed. Each parameter was converted into a binary variable that indicated whether or not the acceptability limit was exceeded at each time point (0 = Acceptability limit not exceeded, 1 = Acceptability limit exceeded). This binary variable (i.e., exceeding the acceptability limits) was set as a positive state for sperm damage. The minimum dose to achieve damage was the optimal cutoff, as it indicates the optimal dose that maximizes the ability to discriminate between samples that had sperm damage (exceeded the acceptability limits for that parameter) and those that had not. The optimal cutoff was determined as the point of the curve closest to (0,1) using Youden’s index [28].

The effect of LPS dose on MDA was analyzed with nonparametric statistics (Kruskal-Wallis and Mann-Whitney tests) while its correlations with the semen quality parameters were assessed with the Spearman correlation coefficient (ρ).

Statistical analyses were performed with SPSS Statistics version 25 (IBM, SPSS Inc., Chicago, IL, USA). We defined *p* ≤ 0.05 as significant.

## 3. Results

### 3.1. Effect of LPS on Sperm Motility

**Figure 2:**
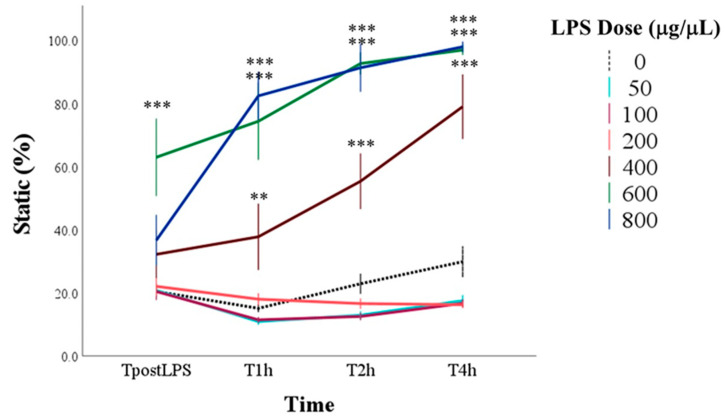
Percentage of static spermatozoa in control samples (0 μg/mL LPS) and in samples with the addition of different doses of LPS evaluated immediately after the inoculation of LPS (TpostLPS), 1 h later (T1h), 2 h later (T2h) and 4 h later (T4h). Values are means and standard errors. ** *p* < 0.01, *** *p* < 0.001 vs. 0 μg/mL LPS (control samples).

Dose, time, and their interaction influenced the percentage of static spermatozoa (for all: *p* < 0.001). In particular, the greatest increases in marginal means compared to control (0 μg/mL LPS) were recorded with doses equal to and greater than 400 μg/mL and from the time T2h. Multiple comparisons showed that at TpostLPS, the group to which 600 μg/mL of LPS was added immediately showed an increase in static spermatozoa compared to the control group. From T1h to T4h, higher percentages of static spermatozoa than the control were found with 400, 600, and 800 μg/mL of LPS (*p* < 0.001; Figure 2).

**Figure 3:**
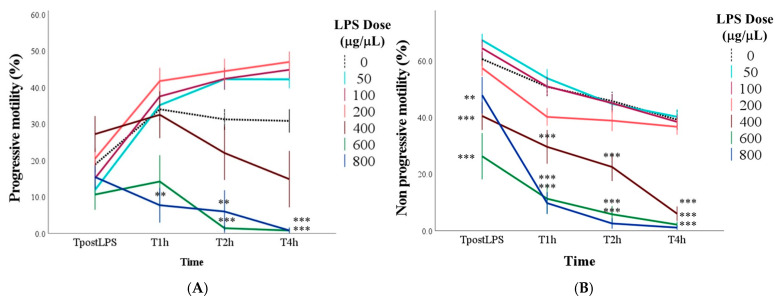
Percentage of progressive motility (Panel (**A**)) and percentage of non-progressive motility (Panel (**B**)) in control samples (0 μg/mL LPS ) and in samples with the addition of different doses of LPS evaluated immediately after the inoculation of LPS (TpostLPS), 1 h later (T1h), 2 h later (T2h) and 4 h later (T4h). Values are means and standard errors. ** *p* < 0.01, *** *p* < 0.001 vs. 0 μg/mL LPS (control samples).

With doses up to 200 μg/mL of LPS, progressive motility showed an increase from TpostLPS to T1 and then stabilized until the last time point (*p* < 0.01; Figure 3A). With doses higher than 200 μg/mL there were no significant changes compared to TpostLPS. After 4 h of incubation, samples with doses greater than 400 μg/mL had a lower percentage of progressive motility compared to the control and, in particular, the mean values were equal to 30.8 ± 3.2% in control samples, 0.8 ± 0.5% with 600 μg/mL, and 0.8 ± 0.8% with 800 μg/mL of LPS (for all: *p* < 0.001; Figure 3A).

The marginal means of percentage of spermatozoa with non-progressive motility gradually decreased over time (*p* < 0.001) and, compared to the control samples, were lower with doses equal to and greater than 400 μg/mL (*p* < 0.001). Notably, with doses of 400, 600, and 800 μg/mL, lower values compared to the control group were found already from the TpostLPS (*p* < 0.01; Figure 3B).

**Figure 4:**
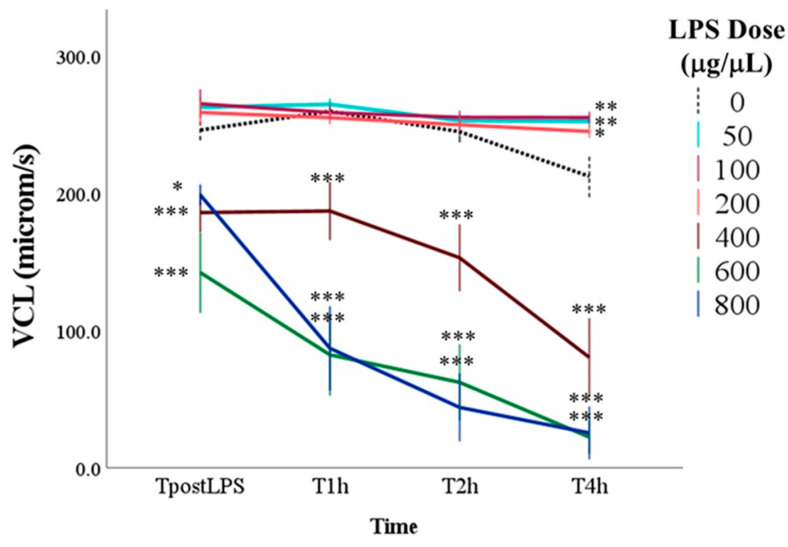
Curvilinear velocity (VCL) in control samples (0 μg/mL LPS) and in samples with the addition of different doses of LPS evaluated immediately after the inoculation of LPS (TpostLPS), 1 h later (T1h), 2 h later (T2h) and 4 h later (T4h). Values are means and standard errors. * *p* < 0.05, ** *p* < 0.01, *** *p* < 0.001 vs. 0 μg/mL LPS (control samples).

The main effect of time, regardless of dose, showed a progressive reduction of VCL over time (*p* < 0.001; Figure 4). The main effect of the dose was highly significant (*p* < 0.001). In particular, the marginal means of samples with doses equal to or greater than 400 μg/mL of LPS were lower than those without LPS (0 μg/mL LPS = 244.3 ± 10.9 μm/s; 400 μg/mL LPS = 155.4 ± 11.8 μm/s; 600 μg/mL LPS = 96.4 ± 12.3 μm/s; 800 μg/mL LPS = 101.8 ± 12.1 μm/s; for all: *p* < 0.001). Multiple comparisons showed that the differences between these samples and the control were significant already immediately after LPS inoculation (TpostLPS) and continued until the last time point (T4h), when samples with 400, 600 and 800 μg/mL of LPS reached values below 100 μm/s (*p* < 0.001). Moreover, at T4h, samples with 50, 100, and 200 μg/mL of LPS showed higher VCL than the control (*p* < 0.05).

**Figure 5:**
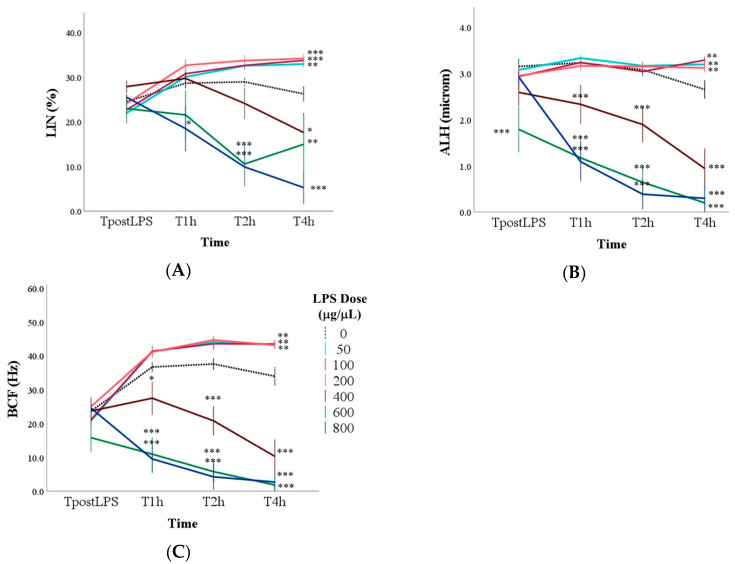
Linearity (LIN, Panel (**A**)), amplitude of lateral head displacement (ALH, Panel (**B**)) and beat cross frequency (BCF, Panel (**C**)) in control samples (0 μg/mL LPS ) and in samples with the addition of different doses of LPS evaluated immediately after the inoculation of LPS (TpostLPS), 1 h later (T1h), 2 h later (T2h), and 4 h later (T4h). Values are means and standard errors. * *p* < 0.05, ** *p* < 0.01, *** *p* < 0.001 vs. 0 μg/mL LPS (control samples).

As for LIN, the main effect of the LPS dose showed a reduction in marginal means compared to the control (27.5 ± 1.7%) only at doses equal to or greater than 600 (20.1 ± 1.9% and 17.2 ± 1.8% with 600 and 800 μg/mL of LPS). In particular, the addition of LPS did not immediately result in a LIN reduction (Figure 5A). At T1h, only the dose of 800 μg/mL of LPS significantly reduced LIN (*p* < 0.05). At T2h, even with 600 μg/mL of LPS, the LIN was reduced compared to the control (*p* < 0.01), while at T4h, lower values than the control were found with the doses of 400, 600 and 800 μg/mL of LPS (*p* < 0.05). On the contrary, samples with doses between 50 and 200 μg/mL of LPS had higher LIN at the last time point than those without LPS (*p* < 0.01; Figure 5A).

Similarly to the other parameters, the marginal means of ALH were lower with 400, 600, and 800 μg/mL of LPS compared to the samples without LPS (*p* < 0.001), with significant differences starting from TpostLPS with 600 μg/mL of LPS, and from T1h for the other two high LPS dosages (*p* < 0.001; Figure 5B).

The BCF confirmed the previous results: The lowest marginal means were found with doses equal to or greater than 400 μg/mL (*p* < 0.001), and multiple comparisons showed that this significant reduction started from T1h (*p* < 0.05; Figure 5C).

Finally, VS.L, VAP, and STR exhibited similar trends to the other kinematic parameters and are presented in Figures S1–S3.

### 3.2. Determination of the Optimal LPS Dose to Induce Spermatic Damage

**Figure 6:**
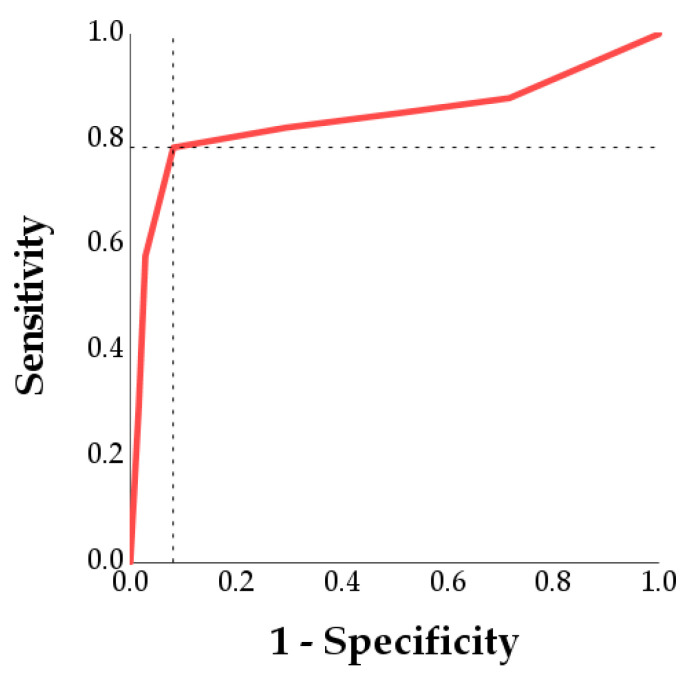
Determination of the minimum dose of LPS to obtain an increase in the percentage of static spermatozoa above the acceptability limit (49.9%) using receiver operating characteristic (ROC) analysis. The point of the curve closest to (0,1) was the threshold (optimal cut-off) and corresponded to 300 μg/mL LPS. At this cut-off, the sensitivity was 0.785 (horizontal dotted line), and the specificity was 0.919 (1-Specificity = 0.081; vertical dotted line).

Table 2 and Table S2 show the limit of acceptability for each parameter according to the means and SD of the control group, as well as a minimal LPS dosage to overcome the acceptability limits and respective sensitivity and specificity. For all parameters, the dosage of LPS that determined the exceeding of the acceptability limits was 300 μg/mL. With this dosage, the sensitivity ranged from 78.5% of the static variable to 90.6% of the VCL. The specificity ranged from 54.1% for progressive motility to 91.9% for static and non-progressive motility. Figure 6 shows, as an example, the determination of the LPS dose threshold for the static cells. The point of the curve closest to 0,1, using Youden’s index, corresponded to the 300 μg/mL LPS; with higher doses, the acceptability limit for static spermatozoa of 41.9% was exceeded with a sensitivity of 0.785 and a specificity of 0.919.

### 3.3. Assessment of Lipid Peroxidation: Malondialdehyde (MDA) Levels

**Figure 7:**
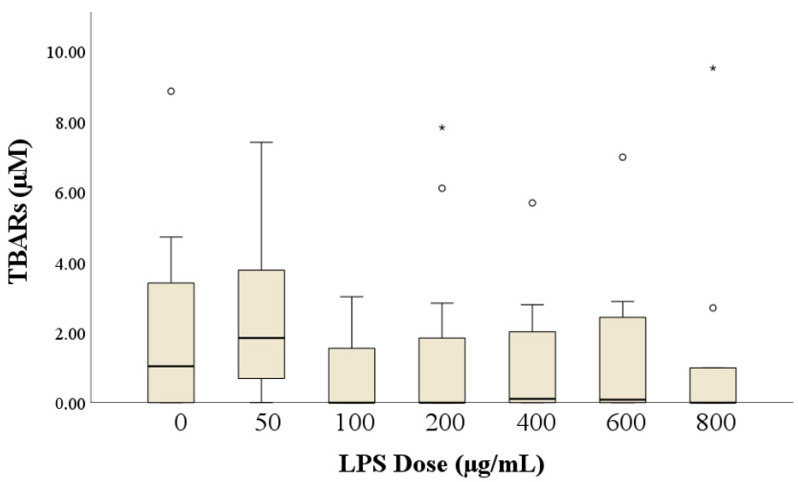
TBARS (thiobarbituric acid reactive substances) levels according to the lipopolysaccharide (LPS) dose. Circles indicate values more than 1.5 × interquartile range below Quartile 1 or above Quartile 3 while asterisks more than 3.0 × interquartile range below Quartile 1 or above Quartile 3.

In more than half of the samples (*n* = 49), the MDA levels were not detectable, and their concentrations were considered as 0. Figure 7 shows the MDA concentrations according to the dose of LPS. The Kruskal–Wallis test showed no effect of LPS, and the multiple comparisons only showed a trend in the difference between 50 and 800 μg/mL LPS (*p* = 0.051). MDA was not correlated with any other semen traits (for all: ρ < |0.3|, *p* > 0.05).

### 3.4. Expression of TLR4 in Rabbit Sperm

**Figure 8:**
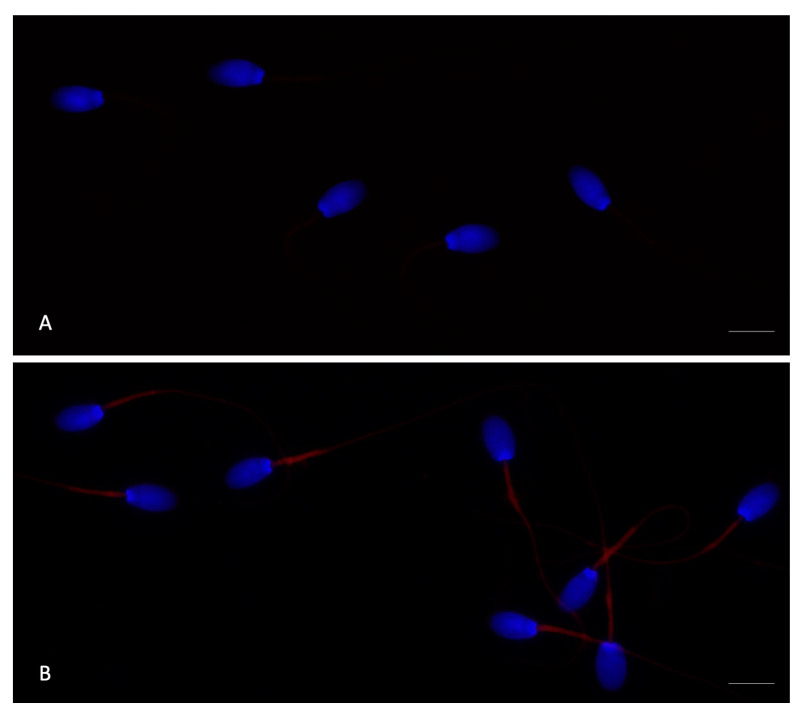
UV (ultraviolet) micrographs of rabbit spermatozoa without (**A**) and after 4 h of incubation with 400 μg/mL LPS (lipopolysaccharide) (**B**) treated with anti-TLR4 antibody. The label is localized in the middle piece of the sperm tail. In (**A**), the TLR4 (Toll-like receptor 4) signal was weak, whereas in (**B**), after LPS incubation, an intense TLR4 signal was observed. Nuclei were stained with DAPI (4′,6-diamidino-2-phenylindole). Scale bars: 5 μm.

Immunofluorescence was performed to examine TLR4 localization in rabbit spermatozoa, both in the absence of LPS (control) and after 4 h of incubation with 400 μg/mL LPS (Figure 8). TLR4 labeling was detected in the middle piece of the tail. Specifically, control spermatozoa exhibited a weak signal, whereas those incubated with LPS displayed a markedly increased TLR4 signal intensity.

## 4. Discussion

This study is the first in vitro investigation to identify the LPS dosage that impairs sperm motility in rabbits. It establishes a dose-dependent threshold for LPS-induced effects, providing a foundation for future research on inflammation-related male infertility in this species. Additionally, we present the first evaluation of TLR4 localization on rabbit sperm, offering new insights into its role in LPS-mediated reproductive alterations.

Most existing studies on rabbits have predominantly examined the effects of bacterial infections on the organs of the male genital tract, with limited investigation into the direct effects of bacteria or their components, such as LPS, on sperm and their mechanisms of action. In this context, Brecchia et al. [6] demonstrated that intraperitoneal administration of 50 μg/kg of body weight of LPS triggers a reversible inflammatory response in rabbit bucks. This inflammatory state persists for approximately 3 days, after which the reproductive activity and the sperm quality are progressively restored. In another study, LPS-induced inflammation in rabbit bucks in vivo resulted in reduced sperm concentration and motility, along with increased ultrastructural alteration of the reproductive tract, observed on days 14–30 post-LPS treatment [3]. In this study, sperm motility was selected as the primary parameter for evaluation due to its critical importance as an indicator of sperm quality [29]. Motility is not only a readily observable and measurable sperm function but also an essential prerequisite for successful fertilization [25,30]. Moreover, numerous studies on rabbits have consistently highlighted a strong correlation between sperm motility and fertility outcomes, underscoring its significance in reproductive performance [31,32,33,34]. Additionally, sperm motility is the most frequently affected parameter in both in vivo and in vitro LPS studies across various animal species [19,20,35]. This decline is recognized as being both time- and dose-dependent, aligning closely with the results observed in our study. Indeed, our findings confirm that both the concentration of LPS and the duration of exposure significantly affected sperm motility, specifically increasing the proportion of static spermatozoa. The most pronounced effect was observed starting with 400 μg/mL, which significantly increased the percentage of static spermatozoa after 2 h of incubation. With the dosage of 600 μg/mL LPS, the detrimental effect on motility was immediate, with a significant increase in static spermatozoa observed immediately following LPS inoculation. This dose-dependent relationship persisted across all evaluated time points (from 1 h to 4 h post-incubation), with the highest LPS concentrations (400, 600, and 800 μg/mL) consistently associated with the greatest proportion of static spermatozoa. Accordingly, both progressive and non-progressive motility of spermatozoa also showed significant changes, which were strongly influenced by the dose and duration of LPS exposure. Surprisingly, progressive motility initially increased from the time of incubation to 1 h post-incubation in samples exposed to LPS doses up to 200 µg/mL, after which it stabilized at subsequent time points. This early increase in progressive motility may be due to a combination of physiological factors, the properties of the TALP medium, and the relatively mild effects of these low LPS concentrations on rabbit sperm [36]. In contrast, after 4 h of incubation, a significant decline in progressive motility was observed in samples treated with LPS doses of 400 µg/mL or higher. Similarly, non-progressive motility gradually decreased over time and was significantly lower in samples treated with doses of 400 μg/mL or higher compared to the control group. Overall, the observed changes indicate a dose–response relationship, with higher dosages of LPS causing more severe and immediate effects on sperm motility. Similar findings have been reported in various species, consistently highlighting the detrimental effects of LPS on sperm motility parameters. However, the dosages required to induce these effects vary significantly between species, reflecting potential species-specific differences in sperm sensitivity to LPS. For example, in rams, Mirshokraei et al. [35] observed that doses of 200 and 400 µg/mL of LPS did not significantly alter the percentage of static spermatozoa after 225 min of incubation, indicating that these concentrations were insufficient to exert a spermicidal effect. Conversely, human sperms [10,18] have shown a much greater sensitivity to LPS. Total and progressive sperm motility were significantly inhibited by LPS at concentrations as low as 0.1–100 µg/mL following incubation periods of 1 to 12 h. Urata et al. [10] further demonstrated a dose-dependent inhibition of motility, with reductions of 15%, 21%, and 50% at doses of 0.1, 1, and 10 μg/mL LPS, respectively, after just 60 min. Similarly, in boars, LPS concentrations of 0.1–10 µg/mL over a 6-h incubation resulted in alterations in motility and increased apoptosis [19]. In mice, even lower concentrations (0.01 μg/mL) reduced sperm motility after 4 h of exposure [20]. This discrepancy highlights potential species-specific differences in sperm sensitivity to LPS, which could be attributed to differences in receptor expression, intracellular signaling mechanisms, and the resilience of sperm to inflammatory stimuli [16,17].

Regarding the sperm kinematic parameters evaluated in our study, curvilinear velocity (VCL) progressively decreased over time, with samples treated with LPS at doses of 400 μg/mL or higher exhibiting significantly lower values. This decline was evident immediately after LPS inoculation and persisted until the final time point (4 h). Interestingly, after 4 h of incubation, samples treated with 50, 100, and 200 μg/mL of LPS displayed higher VCL values than the control group, suggesting a possible stimulatory effect at these lower doses. Finally, linearity (LIN) showed a consistent trend, with reductions observed only at LPS doses of 600 μg/mL or higher. On the contrary, samples with doses between 50 and 200 μg/mL of LPS had higher values after 4 h of incubation than those without LPS. Additionally, the marginal means of the amplitude of lateral head displacement (ALH) were lower in samples exposed to 400, 600, and 800 μg/mL of LPS, indicating, once again, a dose-dependent decline. Similarly, beat-cross frequency (BCF) was lowest at doses of 400 μg/mL or higher. The dose-dependent effects of LPS on sperm kinematic parameters demonstrate that lower doses (50–200 μg/mL) may have a mild stimulatory effect, while higher doses (400 μg/mL and above) significantly reduce sperm velocity. These contrasting effects suggest that LPS modulates sperm function through complex mechanisms, with outcomes varying based on both dose and exposure duration. Previous studies in other species [35,37] have concluded that LPS, at sub-spermicidal concentrations, induces alterations in progressive motility and kinematic parameters that may contribute to infertility. These changes are not attributed to toxicity but are likely mediated by LPS-induced activation of signaling pathways involved in regulating sperm motility [38,39].

The minimum dose of LPS required to reduce sperm motility was determined through ROC analysis, where the acceptability limits were calculated using the control group values and the threshold obtained with Youden’s index. The results fully supported the findings from the Linear Mixed Models analysis: For all the parameters analyzed, the minimum LPS dose that exceeded the acceptability limits was 300 µg/mL.

Among the various mechanisms by which LPS can impair sperm motility, one significant effect is the disruption of intracellular signaling pathways, including a reduction in cAMP levels [18], which are crucial for sperm motility and capacitation [40,41]. Emerging evidence also suggests a direct effect of LPS on sperm through the activation of apoptotic pathways [19]. This effect is mediated through Toll-like receptor 4 (TLR4), the primary receptor for LPS, which has been identified on spermatozoa of various species, including humans [37], rats [20], mice [37], and boars [19]. In these species, TLR4 is localized in the acrosome and tail regions of the spermatozoa [42]. The activation of TLR4 by LPS has been associated with decreased viability, reduced motility, and alterations in the acrosome reaction [43]. In our study, we identified the presence of TLR4 localized in the midpiece of rabbit spermatozoa, marking the first report of its localization in this species. The midpiece of the sperm tail houses mitochondria, essential for energy production and motility [44], suggesting that LPS binding to TLR4 in this region may directly impair mitochondrial function, leading to decreased motility. This finding is particularly noteworthy, as LPS has been shown to disrupt sperm mitochondrial function, leading to impaired ATP production, consequently reducing sperm motility in humans [19,45]. Although direct evidence in rabbits is limited, the conserved nature of TLR4 signaling across species suggests that a similar mechanism may exist in rabbits as well. Notably, the greater overall similarity between rabbit and human TLR4, both in sequence and function, indicates that rabbits may share the same activation pathways, making them a potentially more accurate model for human research in this field compared to other species [46]. Our hypothesis is further supported by the idea that mitochondrial dysfunction plays a crucial role in reduced sperm motility, manifesting as the loss of membrane potential, electron leakage, increased ROS production, and a decreased capacity for energy generation, all of which contribute to impaired motility [45].

Finally, sperm cells are particularly susceptible to oxidative stress due to the high content of polyunsaturated fatty acids (PUFAs) in their membranes and limited antioxidant defenses [47]. This vulnerability can lead to oxidative damage affecting various cellular components, including lipids and DNA. While lipid peroxidation results in the formation of MDA, a marker of oxidative stress, oxidative stress can also cause DNA fragmentation, impairing sperm function and fertility [48]. In our study, we observed no significant correlation between MDA and semen motility parameters, contrasting with previous human findings linking elevated MDA levels to impaired sperm quality [49]. Our finding suggests that, despite the potential for LPS-induced oxidative damage, the antioxidant defense mechanisms in rabbit seminal plasma may effectively mitigate lipid peroxidation of the sperm membranes. Alternatively, oxidative stress in rabbit spermatozoa might primarily induce DNA or mitochondrial damage rather than lipid peroxidation, leading to impaired sperm function without a corresponding increase in MDA levels. This observation aligns with studies indicating that oxidative stress-induced DNA fragmentation can occur independently of lipid peroxidation [49,50]. Additionally, variations in experimental conditions, such as differences in LPS dosages, exposure durations, or pathological factors, might influence the outcomes.

## 5. Conclusions

The identified threshold of 300 µg/mL LPS provides a valuable reference for future research using rabbits aimed at understanding how LPS disrupts sperm function and contributes to male infertility. While primarily relevant to rabbits, these findings have broader implications for reproductive health in other species, including humans, by highlighting the potential link between bacterial infections and infertility. Notably, LPS doses of 50, 100, and 200 µg/mL did not significantly impair sperm motility or kinematic parameters, suggesting that these concentrations likely fall below the threshold required to cause measurable damage to sperm membranes, mitochondria, or motility-related signaling pathways, which are recognized in the existing literature as mechanisms of LPS-induced damage. This resistance may be due to species-specific variations in the localization of LPS receptors on sperm, such as TLR4 [17], or to the intrinsic antioxidant defenses present in seminal plasma [51], which could help counteract the initial effects of LPS at low doses. This study is the first to evaluate Toll-like receptor 4 (TLR4) expression in rabbit spermatozoa, demonstrating its presence in the midpiece of the sperm tail and its responsiveness to LPS in a dose- and time-dependent manner. Furthermore, our study did not reveal a significant correlation between MDA and semen motility parameters, suggesting that LPS-induced motility reduction may be more closely associated with DNA damage or mitochondrial dysfunction rather than lipid peroxidation of sperm membrane lipids. These findings emphasize the need for further studies to clarify the intracellular signaling pathways activated by LPS in spermatozoa and to assess whether these effects are consistent across different species and experimental conditions.

## Figures and Tables

**Table 1:** Definition and description of kinematic parameters evaluated by CASA in rabbit sperm.

Parameter	Description	Definition	Units
Static		Immotile spermatozoa	%
Progressive motility		Progressively motile spermatozoa	%
Non-progressive motility		Spermatozoa that are moving but not progressing forward	%
VCL	Curvilinear velocity	Velocity of the sperm head along its actual curvilinear path	μm/s
LIN	Linearity	Linearity of the curvilinear path (VS.L/VCL ratio)	%
ALH	Amplitude of lateral head displacement	The average value of the extreme side-to-side movement of the sperm head in each beat cycle	μm
BCF	Beat-cross frequency	Frequency of the head crossing the average path trajectory	Hz

**Table 2:** Marginal mean and standard deviation of the control group, as well as the limit of acceptability for each parameter, and minimal LPS dosage to overcome the acceptability limits and respective sensitivity and specificity resulting from the receiver operating characteristic (ROC) analyses.

Parameter	Control		Acceptability Limit	Optimal Minimal LPS Dosage to Overcome the Acceptability Limit	Sensitivity	Specificity
	Mean	Standard Deviation				
Static	22.1%	19.8%	41.9%	300 μg/mL	78.5%	91.9%
Non-progressive motility	49.1%	21.2%	27.97%	300 μg/mL	50.6%	91.9%
Progressive motility	28.8%	18.3%	10.50%	300 μg/mL	90.0%	54.1%
VCL ^1^	240.36	62.61	177.75	300 μg/mL	90.6%	78.6%
LIN ^2^	27.10	7.62	19.48	300 μg/mL	85.1%	55.2%
ALH ^3^	3.03	0.94	2.09	300 μg/mL	89.6%	64.3%
BCF ^4^	32.97	13.02	19.96	300 μg/mL	89.4%	57.8%

^1^ VCL: curvilinear velocity; ^2^ LIN: linearity; ^3^ ALH: amplitude of lateral head displacement; ^4^ BCF: beat-cross frequency.

## Data Availability

The raw data supporting the conclusions of this article will be made available by the authors upon request.

## Supplementary Materials

The following supporting information can be downloaded at: https://www.mdpi.com/article/10.3390/antiox14040431/s1, Table S1: Definition and description of kinematic parameters evaluated using a computer-assisted sperm analysis (CASA) system in rabbit sperm;. Figure S1: Straight line velocity (VSL) in control samples (0 μg/mL LPS) and in samples with the addition of different doses of LPS evaluated immediately after the inoculation of LPS (TpostLPS), one hour later (T1h), two hours later (T2h) and four hours later (T4h). ** *p* < 0.01 and *** *p* < 0.001 vs. 0 μg/mL LPS (control samples); Figure S2: Average path velocity (VAP) in control samples (LPS dose 0) and in samples with the addition of different doses of LPS evaluated immediately after the inoculation of LPS (TpostLPS), one hour later (T1h), two hours later (T2h) and four hours later (T4h). * *p* < 0.05, ** *p* < 0.01, *** *p* < 0.001 vs. LPS Dose 0 μg/mL (control samples); Figure S3: Percentage straightness (STR) in control samples (LPS dose 0) and in samples with the addition of different doses of LPS evaluated immediately after the inoculation of LPS (TpostLPS), one hour later (T1h), two hours later (T2h) and four hours later (T4h). * *p* < 0.05, ** *p* < 0.01, *** *p* < 0.001 vs. LPS Dose 0 μg/mL (control samples); Table S2: Marginal mean and standard deviation of the control group, as well as the limit of acceptability for each parameter, and minimal LPS dosage to overcome the acceptability limits and respective sensitivity and specificity resulting from the receiver operating characteristic (ROC) analyses.
