# Supplementary material for: In Vitro Effects of Lipopolysaccharide on Rabbit Sperm: Toll-like Receptor 4 Expression, Motility, and Oxidative Status
Source: Antioxidants (Basel). 2025 Apr 2;14(4):431. doi: 10.3390/antiox14040431 (PMC12024201; doi:10.3390/antiox14040431)
Supplement: Supplementary file 1 [file antioxidants-14-00431-s001.zip › antioxidants-3526981-supplementary.pdf]

Supplementary material

Table S1. Definition and description of kinematic parameters evaluated using a computer-assisted sperm analysis (CASA) system in rabbit sperm.

Parameter	Description	Definition	Units
**VSL**	Straight line velocity	Velocity of the sperm head along a straight line	µm/s
**VAP**	Average path velocity	Velocity of the sperm head along its average path	µm/s
**STR**	Straightness	Straightness of the path velocity (VSL/VAP ratio)	%

Supplementary Results: Effect of LPS on sperm motility parameters

VSL showed lower marginal means in samples with doses equal to or greater than 400 µg/mL of LPS compared to the control (LPS Dose: 0 µg/mL LPS =68.2±4.0 µm/s; 400 µg/mL LPS=51.8±4.3 µm/s; 600 µg/mL LPS=32.0±4.5 µm/s; 800 µg/mL LPS= 32.8±4.4 µm/s; for all: p<0.001). However, the differences between control and high doses did not start from TpostLPS but from T1h for 600 and 800 µg/mL LPS, and from T2h for samples with 400 µg/mL LPS (p<0.01). Samples with 50, 100, and 200 µg/mL of LPS showed marginal means and values at T4h higher than the control (p<0.001; Figure S1).

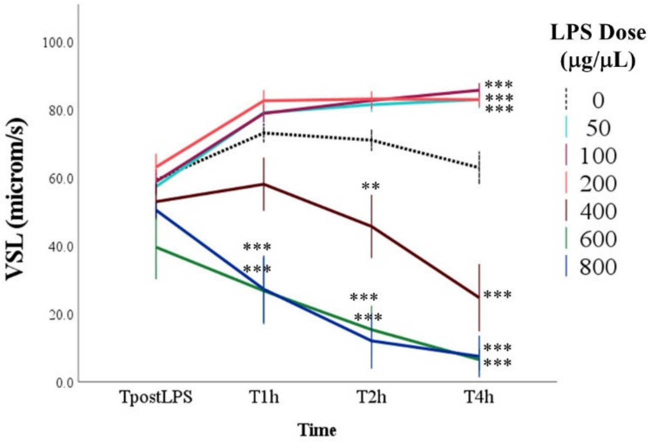

Figure S1. Straight line velocity (VSL) in control samples (0 µg/mL LPS) and in samples with the addition of different doses of LPS evaluated immediately after the inoculation of LPS (TpostLPS), one hour later (T1h), two hours later (T2h) and four hours later (T4h). **p<0.01 and *** p<0.001 vs 0 µg/mL LPS (control samples).

Similarly to the other parameters, VAP was influenced by dose, time, and their interaction (for all: p<0.001). Regardless of the group, its marginal means decreased over time, while the main effect of the LPS Dose showed that the addition of 50, 100, and 200 µg/mL of LPS determined an increase in VAP than control (p<0.01), while the addition of 400 or more µg/mL of LPS reduced it (p<0.001). With 600 and 800 µg/mL LPS, the values were halved compared to the marginal means of the control (47.6±6.4, 50.2±6.3, and 120.9±5.7 for 600, 800, and 0 µg/mL of LPS). Multiple comparisons showed that immediately after inoculation of 400 and 600 µg/mL of LPS (TpostLPS), VAP was decreased compared to the control (p<0.01). From T1h until the end

of observations, doses of 400, 600, and 800 μg/mL determined a reduction in VAP compared to the group without LPS ($p<0.001$). Samples with 50, 100, and 200 μg/mL of LPS showed higher values than the control at T4h ($p<0.05$; Figure S2).

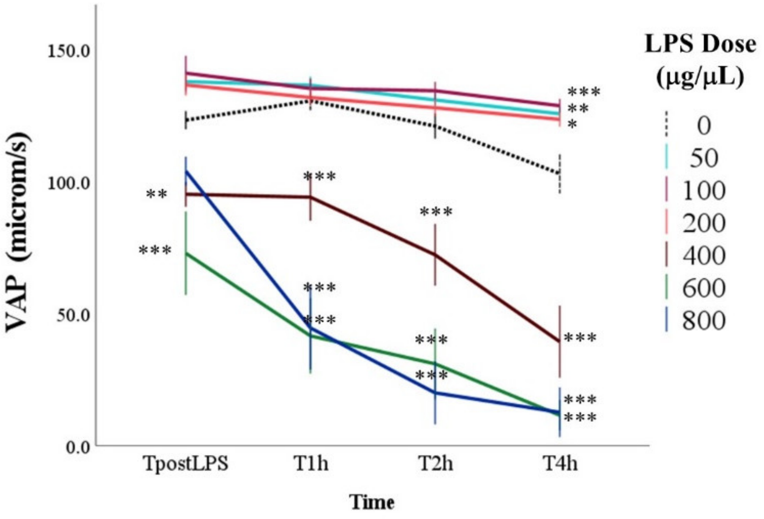

Figure S2. Average path velocity (VAP) in control samples (LPS dose 0) and in samples with the addition of different doses of LPS evaluated immediately after the inoculation of LPS (TpostLPS), one hour later (T1h), two hours later (T2h) and four hours later (T4h). *$p<0.05$, **$p<0.01$, *** $p<0.001$ vs LPS Dose 0 μg/mL (control samples).

STR showed a similar trend: marginal means decreased with doses equal to or greater than 600 μg/mL ($p<0.001$), and multiple comparisons showed differences compared to the control only with the highest dose at T1h (i.e., 800 μg/mL; $p<0.001$), with 600 and 800 μg/mL of LPS at T2h ($p<0.001$), and with 400, 600 and 800 μg/mL of LPS at T4h ($p<0.001$; Fig. S3).

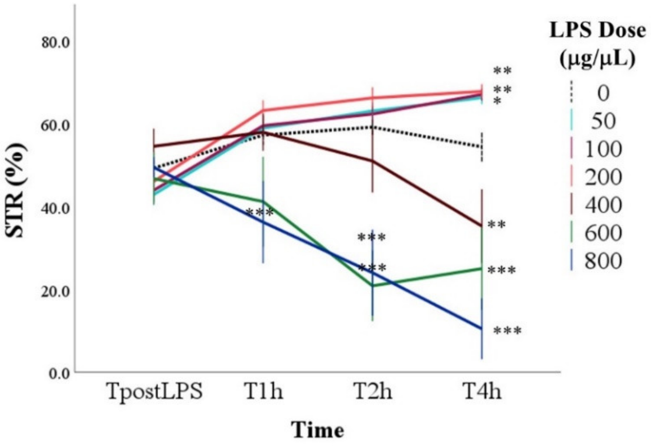

Figure S3. Percentage straightness (STR) in control samples (LPS dose 0) and in samples with the addition of different doses of LPS evaluated immediately after the inoculation of LPS (TpostLPS), one hour later (T1h), two hours later (T2h) and four hours later (T4h). *$p<0.05$, **$p<0.01$, *** $p<0.001$ vs LPS Dose 0 μg/mL (control samples).

Table S2. Marginal mean and standard deviation of the control group, as well as the limit of acceptability for each parameter, and minimal LPS dosage to overcome the acceptability limits and respective sensitivity and specificity resulting from the receiver operating characteristic (ROC) analyses.

Parameter	Control		Acceptability limit	Optimal minimal LPS dosage to overcome the acceptability limit	Sensitivity	Specificity
	Mean	Standard Deviation				
VSL[1]	66.69	21.27	45.42	300 μg/mL	89.6%	63.8%
VAP[2]	119.24	32.68	86.56	300 μg/mL	87.6%	89.2%
STR[3]	55.08	15.87	39.21	300 μg/mL	85.3%	47.7%

[1]VSL: straight line velocity; [2]VAP: average path velocity; [3]STR: straightness.
